# A narrative review of the literature on the pediatric orthopedic management of fibrous dysplasia

**DOI:** 10.3389/fped.2024.1502262

**Published:** 2025-01-31

**Authors:** Elio Paris, Giacomo De Marco, Oscar Vazquez, Christina Steiger, Sana Boudabbous, Romain Dayer, Dimitri Ceroni

**Affiliations:** ^1^ Faculty of Medicine, University of Geneva, Geneva, Switzerland; ^2^ Pediatric Orthopedic Unit, Pediatric Surgery Service, Geneva University Hospitals, Geneva, Switzerland; ^3^ Radiology Department, Geneva University Hospitals, Geneva, Switzerland

**Keywords:** narrative review, pediatric, orthopedic, fibrous dysplasia, bone disorder

## Abstract

Fibrous dysplasia is a congenital, non-inherited, benign intramedullary bone lesion in which the normal bone marrow is replaced by abnormal fibro-osseous tissue. The disorder can be monostotic (involving a single bone) or polyostotic (involving multiple bones). As the abnormal fibro-osseous tissue compromises the mechanical strength of bone, it can result in pain, deformity, fractures, or abnormalities in bone mechanics with inappropriate bone alignment. This narrative review attempts to summarize more than 20 years of observations of patients with FD to help pediatric orthopedists establish a care framework that can improve its identification, understand the impact that endocrinopathies can have on its clinical presentation, and optimize the management of bone disorders. Our focus is specifically on orthopedic manifestations of FD and modern management alternatives. The past 20 years have provided major advances in understanding of fibrous dysplasia (FD), and it is clear that the pediatric orthopedist's role remains highly relevant in the management of all types of FD. Surgical treatment remains appropriate when pain is unresponsive to other medical treatments, when a pathological fracture is impending or has happened, when a deformity is worsening or has formed, or when there is a suspicion of malignant transformation. The pediatric orthopedist must be aware, therefore, of the particularities of the different bones on which they may be called to intervene, and they should give very careful consideration to their operative strategy, which must be adjusted to the biological and static characteristics of the bone.

## 1 Introduction

Fibrous dysplasia (FD) is a non-hereditary benign genetic bone disorder presenting as either an isolated skeletal lesion (its monostotic form) or affecting multiple bones (its polyostotic form) (1). FD can be associated with single or multiple endocrinopathies, such as hyperthyroidism, hyperparathyroidism, acromegaly, diabetes mellitus, and Cushing syndrome (2). More rarely, FD can be accompanied by one or more soft-tissue myxomas (3). Finally, FD can present in a syndromic form involving polyostotic FD, precocious puberty in females, and café-au-lait spots (McCune–Albright syndrome) (4). Treating FD thus requires multidisciplinary care that brings pediatricians, endocrinologists, radiologists, metabolic bone specialists, pathologists, geneticists, ophthalmologists, neurosurgeons, otolaryngologists, physiotherapists, and pediatric orthopedists together at the child's bedside (5). However, pediatric orthopedists are often the first to be confronted with FD, before it has even been diagnosed, either through dealing with bone pain, bone-length inequality, bone deformity, or, far more frequently, a pathological fracture. Thus, a personalized approach to patient care is recommended, one that depends on every member of the care team (6).

This narrative review attempts to summarize more than 20 years of observations of patients with FD to help pediatric orthopedists establish a care framework that can improve its identification, understand the impact that endocrinopathies can have on its clinical presentation, and optimize the management of bone disorders. Our focus is specifically on orthopedic manifestations of FD and modern management alternatives.

## 2 Historical review

Hamartomatous fibro-osseous diseases of the bone fascinated famous names in medicine for a long time, giving rise to countless descriptive speculations. In 1864, Gerhard Engel wrote about “a case of cystoid degeneration of the entire skeleton”. In 1891, Friedrich Daniel von Recklinghausen examined the same case's pathology and anatomy in detail, and he defined it using the term *osteitis fibrosa generalisata* (7). Its causation remained unknown, but establishing *osteotis fibrosa* as a disease marked the beginning of knowledge regarding its etiology. Recklinghausen hypothesised that *osteitis fibrosa generalisata* and *osteitis deformans*, first observed by Paget in 1876, were two different manifestations of similar diseases (8). Since then, *osteitis fibrosis cystica*, also known as *osteitis fibrosa*, *osteodystrophia fibrosa*, and von Recklinghausen's disease of bone, has been recognized as being caused by hyperparathyroidism. In 1937, Albright et al. described five cases characterized by bone lesions that tended to be unilateral and whose histological appearance was suggestive of *osteitis fibrosa* (4). Their description mentioned that patients also presented with brown, non-elevated, pigmented areas of the skin that tended to be on the same side as the bone lesions, together with an endocrine dysfunction, which, in females, was associated with precocious puberty (4). The term *osteitis fibrosa disseminata* was then suggested to differentiate this pathology from the *osteitis fibrosa generalisata* resulting from parathyroidism and from *osteitis fibrosa localisata* (a solitary bone cyst). In 1938, Jaffe drew Lichtenstein's attention to eight subjects presenting with multiple osseous lesions and sufficient features in common to class them as a distinct clinical pathology, which was attributed the name of “polyostotic fibrous dysplasia’ (9). Lichtenstein and Jaffe introduced the term “*fibrous dysplasia*” in 1942, emphasizing that the condition could occur in monostotic form (in a single bone) or polyostotic form (in multiple bones) (10). They also insisted on the fact that “the graver cases could present abnormal pigmentation of skin, premature sexual development, hyperthyroidism or still other extraskeletal abnormalities” (10). However, in 1922, it was Weil who first described the clinical picture known as McCune–Albright syndrome and defined it as involving polyostotic FD, precocious puberty in females (but not in males), and café-au-lait spots on the skin (11). Since Albright was the first to establish this syndrome as a clinical pathology, separate from other recognized osseous diseases, Gorham et al. preferred to associate the condition with Albright's name in 1941 (12). However, history added McCune's name to the syndrome as, in 1937, he reported a rather similar case who presented with *osteodystrophia fibrosa* combined with precocious puberty, pathological pigmentation of the skin, and hyperthyroidism (13). FD has since been reported in association with several endocrinopathies, such as hyperthyroidism, hyperparathyroidism, acromegaly, diabetes mellitus, and Cushing syndrome (2). Finally, a rarer but well-described disorder, characterized by FD in single or multiple bones and associated with one or more soft-tissue myxomas, has also been described and is called Mazabraud's syndrome (3).

## 3 Epidemiology

The epidemiology of FD remains poorly understood, not only because it is rare but also because mild, asymptomatic FD often goes undiagnosed (14). Furthermore, research on FD is mostly conducted in university hospitals, where bias is very likely, due to the preponderance of severe cases that could inhibit accurate epidemiological measurements (15). FD is currently thought to constitute 2.5% of all bony lesions and 5%–7% of all benign lesions (16). Its incidence has been estimated to be in the 1 in 4,000 to 1 in 10,000 range (15, 17). Only one cohort study based on a nationwide registry has been performed, in Denmark, and it demonstrated an incidence of FD of 3.6 cases per 1,000,000 person-year, with a prevalence of the disease of 61 cases per 1,000,000 people, corresponding to1 case in 16,500 people (15). Another estimation of FD's prevalence cites a rate of 1 in 30,000 (18).

FD affects both sexes equally and is most commonly first diagnosed in children or young adults. Age at diagnosis is commonly between the ages of 5 and 30 years old, or more usually between 11 and 20 (15). The monostotic form represents approximately 70%–80% of cases and is usually diagnosed between 10 and 30 years old. The polyostotic form represents 20%–30% of cases, with most patients becoming symptomatic before 10 years old (19). In some series, the monostotic form is 7.6 times more frequent than the polyostotic form (20). Finally, the prevalence of McCune–Albright syndrome is estimated to be from 1 in 100,000 to 1 in 1,000,000 (21).

Lesion location is closely correlated with the clinical form of FD. Indeed, the most common sites of skeletal involvement in monostotic FD are ribs, the proximal femur, and the craniopharyngeal bones, particularly the posterior maxilla (22–24). Lesions usually only involve a small segment of the affected bone, even if FD is present along its complete length. Polyostotic FD is most often encountered in the femur, tibia, pelvis, and foot; less commonly affected sites include the ribs, skull, and bones of the upper extremity, with the cervical and lumbar spine and the clavicles rarely being affected (25).

As a rule, when monostotic and polyostotic FD affect long bones—the tibia, femur, and humerus—they typically occur in the diaphysis or metaphysis. Several authors have postulated that FD bone lesions usually spare the epiphysis (4, 26–28) and, when present, epiphyseal involvement depends primarily on the patient's age. Indeed, the literature suggests that diaphyseal and metaphyseal lesions can progress with growth and, in adults, can result in the involvement of the epiphysis after physeal closure (26–30). On the contrary, cases of FD with epiphyseal involvement before puberty are quite rare (4, 29, 31–34).

## 4 Etiopathogenesis

FD is a rare, non-inheritable, genetic bone disease caused by a postzygotic activating mutation in an important bone-forming gene known as *GNAS*, the guanine nucleotide-binding alpha stimulating complex locus, which is situated in chromosome 20q13.3 and encodes the alpha subunit of the heterotrimeric Gs protein (14). As a reminder, postzygotic mutation is a change in the genome that is acquired during the patient's lifespan instead of being inherited from their parent(s) through the fusion of two haploid gametes. It follows that the chronological timing of the mutation will be responsible for a somatic mosaicism, determining both the severity of the affection and contributing to its clinical heterogeneity (35). Mutations occurring earlier, during the embryonic development phase, are expected to cause extensive skeletal involvement. Moreover, earlier occurrences can result in the mutation's expression in other cell types, such as endocrine cells and melanocytes, subsequently resulting in the clinical characteristics of McCune–Albright syndrome, which combines bone lesions, endocrine dysfunctions, and café-au-lait pigmentation spots (35). Contrarily, mutations occurring later in fetal development will result in fewer mutated cells, but their dispersal will nevertheless contribute to developing the multiple bone lesion locations identifiable as polyostotic FD. Finally, when the mutation occurs postnatally, its expression in the more committed cell-lineage progenies leads to a confined, localized expression of the disease known as monostotic FD. In short, mutations that occur at early stages of embryogenesis typically result in a widespread distribution of bone lesions (1). Conversely, mutations occurring in later stages result in a more localized distribution of the disease (1). This also helps to understand why the monostotic form of FD never progresses to the polyostotic form. Furthermore, mutant cells appear to decrease in number over time, resulting in a gradual cessation of the lesion's growth (36). This phenomenon explains the age-related changes in the tumor's progression rate and any observed stabilization of FD, typically after adolescence.

In the bone tissues, the activating mutation of the alpha subunit of the heterotrimeric Gs protein results in an inappropriate overproduction of cyclic adenosine monophosphate (cAMP) in the affected cells of the osteogenic lineage (14). This, in turn, leads to the accelerated production of bone marrow stromal cells and the simultaneous inhibition of the differentiation of these progenitor cells into mature osteoblasts (37). These immature, dysfunctional cells give rise to the anarchic production of under-mineralized fibro-osseous tissue with a disturbed and thus poor-quality micro-architecture. In addition, in the extensive disease, this mineralization defect is exacerbated by the increased fibroblast growth factor 23 expression of the mass of mutated osteogenic cells, leading to renal phosphate wasting and impaired 1.25 vitamin D production (38). Finally, a localized increase in osteoclastogenesis, in and around FD lesions, has been noted and seems to constitute a further pathophysiological characteristic of the disease, leading to a local increase in bone resorption (38). This phenomenon is believed to be due to the significant up-regulation of the receptor activator of nuclear factor κ-B ligand (RANKL) by GNAS-mutated osteogenic cells and their increased production of interleukin-6 (IL-6) (37, 39). The increased number and clustering of osteoclasts give rise to a tunneling resorption pattern and endosteal fibrosis analogous to the changes characteristically observed in hyperparathyroidism (38). In summary, the phenomena which govern FD induce a localized increase in bone turnover, with increased deposition of abnormal, under-mineralized fibro-osseous tissue that leads to pain, the further expansion of FD lesions, and an increased risk of deformities and fractures.

## 5 Natural history

Pediatric orthopedists must have some knowledge about FD's natural history in order to be able to rationally present an indication for possible orthopedic treatment. It is crucial, therefore, to remember that the chronology of the mutation's appearance is responsible for a mosaicism that determines the disease's extent and clinical manifestations (40, 41). Thus, FD can appear in several phenotypes along a continuum of clinical presentation, with monostotic FD at one end, McCune–Albright syndrome at the other, and polyostotic FD in the middle. Monostotic FD could even be considered as a rough shape of McCune–Albright syndrome (42).

Regarding the occurrence of lesions, it is important to emphasize that after 6 years old, all the areas potentially affected by FD will be detectable, and thus no new foci of FD—certainly no new areas of clinical significance—will subsequently appear. The situation is slightly different regarding the evolution of FD lesions. Monostotic lesions may moderately expand in size during skeletal growth, but they typically lose the potential to develop and become biologically inactive and, therefore, quiescent after maturity. Polyostotic lesions, for their part, usually continue to progress, even in adulthood and long after skeletal maturity (43–45).

However, there is a current view that FD lesions tend to burn out with age; this idea is supported by some evidence demonstrating that there is a significant drop in the number of mutation-bearing cells among children with FD as they age, a phenomenon which can be accompanied by the emergence of areas of bone which appear histologically normal (46). This effect is probably due to the increased apoptosis of mutant skeletal progenitor cells, concomitant normal progenitor cell multiplication and the resulting renovation and formation of normal skeletal bone (46, 47). It therefore follows, in this view, that fibrous lesions tend to become more sclerotic with age (46, 47).

The progressive replacement of normal bone and marrow with excessive fibrous tissue leads to a failure of immature bone to remodel into mature lamellar bone, which can result in pathological fractures and inappropriate bone alignment in response to mechanical stresses (31). It is thus the weight-bearing long bones that are more subject to fractures. These fractures can be major or appear as microfractures that progressively deform the bone. These bone deformations themselves may subsequently alter bone stability and promote the occurrence of new fractures due to structural insufficiency—a sort of vicious circle. Nevertheless, dysplastic bone seems to support an entirely coherent healing process, even if it cannot eliminate the foci of FD, and this can lead to repeated fractures in the same location.

Finally, it is important to note that some FD lesions may transform, over time, into either benign or malignant tumors. Firstly, it is recognized that transformation into an aneurysmal bone cyst (ABC) can occur in any bone with FD, since ABC can develop in many pre-existing benign bone tumors (48, 49). Usually, an ABC in a bone with FD is accompanied by growing cysts filled with blood that develop out of the fibrodysplastic bone. The cystic lesion typically expands much more rapidly than FD would, potentially leading to a weakening of the bone and a subsequent fracture. More worrying is the cystic degeneration of FD lesions, in which a locally aggressive form of the disease evolves insidiously into a cystic lesion involving cortical disruption and soft tissue extension, leading to a high risk of pathological fracture (50). Finally, the malignant degeneration of FD lesions is rare but can occur; the most common malignancies are osteosarcoma, fibrosarcoma, and chondrosarcoma (16, 51). Most reported cases appeared to be associated with radiation therapy, which is no longer used to manage FD. But malignancies arising from FD lesions can also occur with no history of radiation, and any change in perceived pain or sudden worsening of an FD lesion should alert the physician to a potential malignant transformation (52).

## 6 Clinical presentation

It is very common for pediatric orthopedists to be the first specialist consulted for FD, whatever its form, even though some children with McCune–Albright syndrome are referred to other specialists because of their non-orthopedic symptoms, such as skin pigmentation or precocious puberty. Patients' monostotic lesions are very frequently diagnosed incidentally on radiographs taken for unrelated symptoms and then urgently referred to an orthopedic specialist, even if they are asymptomatic. Monostotic lesions are usually diagnosed in the second or sometimes the third decade of life (53, 54). Even in the lower limbs, monostotic lesions have a low fracture risk and may, therefore, be observed without a specific need for intervention. The frequency with which patients with monostotic lesions should be re-examined depends on symptoms, age, the extent of the lesion, and, above all, the presence of angular deformity. Children with polyostotic FD will be referred to a specialist at an earlier age due to pain, a limp, a lower-limb-length discrepancy, or a deformity. A diagnosis of FD is often made when a pathological fracture occurs. It should be remembered, however, that the initial extent of bone involvement may be misleading as small lesions can escape detection by the initial bone scan, and many lesions will subsequently expand in young children. The proximal part of the femur is a very common site of polyostotic FD as it is the anatomical region where lesions result in the greatest functional impairment (47, 55–57). Deformities typically occur in the frontal plane, ranging from coxa vara to the well-known 'shepherd's crook' deformity. Clinicians should not underestimate other deformities, however, such as a retroversion or a flexion deformity of the proximal femur that can compromise the hip's bone structure and biomechanics. Scoliosis is also frequent in FD, and it can lead to potentially devastating complications if unrecognized and untreated (58, 59).

## 7 Radiological investigations

When a pediatric orthopedist is first confronted with a child with a suspected FD lesion, several questions come to mind, and certain radiological investigations will provide useful guidance for subsequent treatment. Plain radiography, computed tomography (CT), magnetic resonance imaging (MRI), and bone scintigraphy are the most frequently used technologies for evaluating FD.

On plain x-rays, a classic FD lesion area in the axial skeleton has a radiolucent “ground-glass” appearance, usually smooth and homogeneous, but not centrally located within medullary bone. The lesion usually causes cortical thinning due to enlarged fibro-osseous masses within the bone, leading to endosteal scalloping. A periosteal reaction is usually absent unless the lesion is associated with a pathological fracture. Even if endosteal scalloping is present, a smooth outer cortical contour is always maintained, although the lesion may undergo expansive remodeling secondary to the enlarging mass of fibro-osseous tissue. Small islands of cartilage are also visible, and these may later ossify and be seen as dense punctate or flocculent calcifications within FD lesions (60).

The orthopedist's first concern should be whether the bone damage is isolated or multiple bones are affected. Bone scanning using technetium 99 m methyl diphosphonate (Tc-99 m MDP) as an imaging agent has been used for a very long time to detect metabolically active lesions and assess the extent of FD, especially in young patients. However, it must be kept in mind that the small foci of FD may escape detection in an initial bone scan, especially if the child is younger than 6 years old. Other imaging techniques for assessing the extent of the disease have grown in importance over the past few years, such as whole-body MRI and positron emission tomography (PET). MRI has a high specificity and sensitivity for detecting focal bone lesions and bone marrow infiltration, even before the mineralized bone has been destroyed. PET is a combination of nuclear medicine and biochemical analysis that measures the metabolic activity of the cells in the body tissue being explored. PET detects metabolization, whereas other types of nuclear medicine examinations detect the amount of a radioactive substance collected in body tissue to examine its function. Using PET, FD lesions typically show varying uptakes of 18-F-fluorodeoxyglucose (18-F-FDG) and 18-F-sodium fluoride (18-F-NaF) (57, 61). Thus, PET can detect biochemical changes in tissue that help to identify the onset of a disease process before anatomical changes related to the disease can be seen using other imaging techniques like CT or MRI (1).

Once the diagnosis has been made and the extent of FD lesions has been documented, questions arise concerning which imaging technique should be used to monitor them and how often. Radiographs are used selectively to monitor lesion progression after the initial diagnostic bone scans, and a follow-up bone scan is not recommended. Newer imaging technologies, such as low-dose 2-D or 3-D radiography, have now replaced the traditional methods, and in many institutions, low-dose lower extremity imaging (i.e., EOS) is routinely performed to monitor the evolution of all newly identified cases of FD (62).

Some lesions need to be investigated more precisely, and CT imaging, which better delineates morphological changes in bone, is the technique of choice, superior to radiographs (63). CT can define the anatomy of individual lesions, the extent of disease, the degree of cortical thinning, and any associated deformities far more precisely than plain radiographs. CT scans can also identify soft tissue masses and bone destruction, and, in many cases, they can suggest malignant transformations. For pediatric orthopedists, CT is the most suitable imaging technique for the preoperative planning of surgery on deformities due to FD, particularly at the level of the proximal femur. CT-based 3-D modeling is also becoming more accessible, allowing for better characterization of the deformity and better planning of corrective surgery.

Since FD lesions are clearly distinguishable on MRI, this radiological investigation remains very useful. It is also helpful for evaluating complex cases of FD, such as in patients with compressed neurological structures in the spinal canal. MRI also enables better recognition of the epiphyseal lesions that can lead to severe joint deterioration. It can also be useful for assessing malignant change and showing whether tumors extend into surrounding soft tissues. Indeed, diffusion-weighted imaging (DWI) can help to differentiate benign lesions from malignant osseous lesions. Finally, MRI is the technique of choice for detecting the cystic degeneration of FD lesions or their transformation into ABCs, with all the negative repercussions such changes can have on bone stability.

## 8 Surgical management

The surgical management of FD of the bone remains a real challenge for pediatric orthopedic surgeons: its vast clinical spectrum involves variable lesion sizes and locations, significant potential for lesion evolution, and, above all, a huge impact on bone stability. The surgical treatment of FD principally seeks to manage uncontrollable bone pain, care for impending or historic pathological fractures, correct deformities, and look out for any potential malignant transformations (64). The management of FD in growing children is more complex as lesions are more likely to progress and the disease may disrupt bone growth. Thus, there is a host of parameters and principles that pediatric orthopedists must keep in mind before proposing treatment.

1. It is important to remember that there is no spontaneous resolution of FD, just as there is no cure. The best that can be hoped for is that fibrous lesions will become more sclerotic with age (46, 47, 57, 58) as apoptosis of mutant skeletal progenitor cells increases with the passing years. Thus, there are only rare indications for non-operative orthopedic management in the hope that the disease will be attenuated.
2. It is also important to keep in mind that the diagnosis of FD is mainly based on a clinical examination and typical radiographic features (65). Thus, if the imaging features are characteristic, the lesion does not require histology (1, 66). Tissue biopsy is thus reserved for cases of radiological uncertainty and when concern for secondary malignancy requires strict assessment (67).
3. It is recognized that the different forms of FD do not behave in a similar way. The patient's age is also a key factor in determining how lesions evolve. Monostotic lesions usually lose the potential to progress and become quiescent and biologically inactive after skeletal maturity (68). Conversely, polyostotic lesions are progressive in children and may even continue to progress in adulthood (43).
4. It is illusory to believe that intralesional curettage or even excision of the affected bone can remove all the mutant cells; therefore, one should expect new FD tissue to grow into that area, even when a graft has been used (47).
5. Worse still, it is currently accepted that regardless of the type or volume of a bone graft, the extent of the FD or the patient's age, a graft will sooner or later be resorbed by the dysplastic lesion (62). Some synthetic bone grafts, such as tricalcium phosphate or calcium phosphate cement, probably take the longest to be resorbed into the lesion. However, there may be exceptional indications for using an allograft in conjunction with internal stabilization, since the graft material may provide a temporary, unexpected stability's augmentation for the internal fixation (42).
6. The underlying bone weakness occurring in FD must be considered a constant and be integrated into the therapeutic process. Indeed, treating fractures with casts and non-weight-bearing management should be avoided or at least well-thought-out. Prolonged non-weight-bearing treatments, with or without cast immobilization, will irremediably aggravate any pre-existing bone fragility (68).
7. Usually, remodeling of angulation can not occur, and, when present, deformities may even give rise to recurrent fractures, leading to an accentuation of those deformities (67). Thus, any residual deformities should be avoided as much as possible and, when present, they should be corrected at all costs.
8. Prophylactic treatment makes sense in FD, especially in the context of impending pathological fractures. Prophylactic treatment of long bone lesions should be considered for patients with chronic weight-bearing pain, and for lesions with an axial length >30 mm, with important cortical thinning and bony expansion, with circumferential cortical involvement >50%, and in the presence of cystic degeneration or an ABC transformation (67, 68).
9. Choosing the right osteosynthesis material constitutes a subject of controversy in itself. Pediatric orthopedists treating either a pathological fracture or a deformity should think in terms of permanent stabilization. This implies that it may be necessary to either change the osteosynthesis material if growth is still significant or resort to a definitive bone fixation.
10. Plates and screws have been used to treat FD for decades. However, in many cases, the cortex of the affected bone may be severely compromised, and this may lead screws implanted in deficient fibro-osseous tissue or FD lesions to lose stability, and fixation may subsequently fail. Thus, using a typical plate and screw device should be discouraged unless a sufficient number of screws can be placed outside the FD lesions. Plate and screws nevertheless remain an excellent option for bridging a monostotic and localized lesion, and they should provide adequate mechanical stability.
  Much stricter rules govern the use of plate and screw constructs in cases of FD (64).
  a. Locking plates with blocked screws to the thread holes are recommended to act as an internal fixator.
  b. The longest possible plate able to cover the whole bone should be selected, particularly in polyostotic lesions.
  c. A maximum number of screws should be used since the construction adds significantly to the total pull-out strength.
  d. Finally, titanium should be preferred over stainless steel since its elastic modulus is closer to that of normal bone.
11. Intramedullary devices seem to be the current gold standard for the fixation of long bone lesions due to FD, both because of their load-sharing properties and their satisfactory results (64, 69). In skeletally immature children, elastic nails can be a temporary solution, even if they do not provide the same stability as rigid nails. Elastic nails can also be an interesting therapeutic solution for the upper limbs, which are not subject to an axial load (62). The longest nail possible should be selected (62, 69). The femur is a special case for which a cervico-diaphyseal configuration with one or two fixation points in the femoral neck should be recommended (62, 69) in order to prevent a typical varus deformity of the neck (53, 56, 70).
12. The correction of deformities deserves its own chapter, as their frequent occurrence in three planes means that they can be severe and challenging to correct. Accurate preoperative planning is essential to minimize the hazards inherent in this procedure. Three-dimensional reconstruction images can prove very useful or even essential to fully understand the deformity and to plan its correction (62). 3-D printed models are an excellent new tool for preoperative planning, simulating the procedure on printed bones, and providing intraoperative guidance (62). Ideally, everything should be done so that the deformity can be corrected using a single wedge-resection osteotomy (62).

## 9 Pharmacologic treatment

To date, there is no approved curative therapy for this disorder, and only surgery can significantly change the future of patients with FD (62). Bisphosphonates (BP) had raised a lot of hopes and have been used widely, but there is no evidence that they produce a significant effect on the course of the disorder. Even if BP are recognized to decrease osteoclastic activity and bone turnover, and despite their widespread use over the last 20 years in FD, there is still no consensus on their indications, dosage, and their route of administration (62). About this, the food and drug administration (FDA) still considers to date FD an off-label indication for BP treatment. Only pain management and high bone turnover (as measured by serum biomarkers) in polyostotic fibrous dysplasia may be credible indications for BP treatment (6). To date, there is unfortunately no research focus to improve medical therapy of polyostotic fibrous dysplasia, and it is very unlikely that any new therapy would be found in the near future.

## 10 Conclusions

The past 20 years have provided us with major advances in our understanding of fibrous dysplasia (FD). Ongoing genetic research may lead to a more precise understanding of the exact gene mutations involved in FD, allowing physicians to develop more effective non-surgical treatments. To date, there is no treatment that can cure FD, but newer generations of medications, such as bisphosphonates and denosumab, have opened up new perspectives. Greater experience with these medications may also enable us to provide more effective treatments. The pediatric orthopedist's role remains highly relevant in the management of all types of FD. Indeed, surgical treatment remains appropriate when pain is unresponsive to other medical treatments, when a pathological fracture is impending or has happened, when a deformity is worsening or has formed, or when there is a suspicion of malignant transformation. The pediatric orthopedist must be aware, therefore, of the particularities of the different bones on which they may be called to intervene, and they should give very careful consideration to their operative strategy, which must be adjusted to the biological and static characteristics of the bone.
